# Training a Deep Contextualized Language Model for International Classification of Diseases, 10th Revision Classification via Federated Learning: Model Development and Validation Study

**DOI:** 10.2196/41342

**Published:** 2022-11-10

**Authors:** Pei-Fu Chen, Tai-Liang He, Sheng-Che Lin, Yuan-Chia Chu, Chen-Tsung Kuo, Feipei Lai, Ssu-Ming Wang, Wan-Xuan Zhu, Kuan-Chih Chen, Lu-Cheng Kuo, Fang-Ming Hung, Yu-Cheng Lin, I-Chang Tsai, Chi-Hao Chiu, Shu-Chih Chang, Chi-Yu Yang

**Affiliations:** 1 Graduate Institute of Biomedical Electronics and Bioinformatics National Taiwan University Taipei Taiwan; 2 Department of Anesthesiology Far Eastern Memorial Hospital New Taipei City Taiwan; 3 Department of Computer Science and Information Engineering National Taiwan University Taipei Taiwan; 4 Department of Information Management Taipei Veterans General Hospital Taipei City Taiwan; 5 Medical Artificial Intelligence Development Center Taipei Veterans General Hospital Taipei City Taiwan; 6 Department of Information Management National Taipei University of Nursing and Health Sciences Taipei City Taiwan; 7 Department of Electrical Engineering National Taiwan University Taipei Taiwan; 8 Graduate Institute of Networking and Multimedia National Taiwan University Taipei Taiwan; 9 Department of Internal Medicine Far Eastern Memorial Hospital New Taipei City Taiwan; 10 Department of Internal Medicine National Taiwan University Hospital National Taiwan University College of Medicine Taipei Taiwan; 11 Department of Medical Affairs Far Eastern Memorial Hospital New Taipei City Taiwan; 12 Department of Surgical Intensive Care Unit Far Eastern Memorial Hospital New Taipei City Taiwan; 13 Department of Pediatrics Far Eastern Memorial Hospital New Taipei City Taiwan; 14 School of Medicine National Yang Ming Chiao Tung University Taipei Taiwan; 15 Artificial Intelligence Center Far Eastern Memorial Hospital New Taipei City Taiwan; 16 Section of Health Insurance Department of Medical Affairs Far Eastern Memorial Hospital New Taipei City Taiwan; 17 Medical Records Department Far Eastern Memorial Hospital New Taipei City Taiwan; 18 Department of Information Technology Far Eastern Memorial Hospital New Taipei City Taiwan; 19 Section of Cardiovascular Medicine Cardiovascular Center Far Eastern Memorial Hospital New Taipei City Taiwan

**Keywords:** federated learning, International Classification of Diseases, machine learning, natural language processing, multilabel text classification

## Abstract

**Background:**

The automatic coding of clinical text documents by using the *International Classification of Diseases, 10th Revision* (ICD-10) can be performed for statistical analyses and reimbursements. With the development of natural language processing models, new transformer architectures with attention mechanisms have outperformed previous models. Although multicenter training may increase a model’s performance and external validity, the privacy of clinical documents should be protected. We used federated learning to train a model with multicenter data, without sharing data per se.

**Objective:**

This study aims to train a classification model via federated learning for ICD-10 multilabel classification.

**Methods:**

Text data from discharge notes in electronic medical records were collected from the following three medical centers: Far Eastern Memorial Hospital, National Taiwan University Hospital, and Taipei Veterans General Hospital. After comparing the performance of different variants of bidirectional encoder representations from transformers (BERT), PubMedBERT was chosen for the word embeddings. With regard to preprocessing, the nonalphanumeric characters were retained because the model’s performance decreased after the removal of these characters. To explain the outputs of our model, we added a label attention mechanism to the model architecture. The model was trained with data from each of the three hospitals separately and via federated learning. The models trained via federated learning and the models trained with local data were compared on a testing set that was composed of data from the three hospitals. The micro *F*_1_ score was used to evaluate model performance across all 3 centers.

**Results:**

The *F*_1_ scores of PubMedBERT, RoBERTa (Robustly Optimized BERT Pretraining Approach), ClinicalBERT, and BioBERT (BERT for Biomedical Text Mining) were 0.735, 0.692, 0.711, and 0.721, respectively. The *F*_1_ score of the model that retained nonalphanumeric characters was 0.8120, whereas the *F*_1_ score after removing these characters was 0.7875—a decrease of 0.0245 (3.11%). The *F*_1_ scores on the testing set were 0.6142, 0.4472, 0.5353, and 0.2522 for the federated learning, Far Eastern Memorial Hospital, National Taiwan University Hospital, and Taipei Veterans General Hospital models, respectively. The explainable predictions were displayed with highlighted input words via the label attention architecture.

**Conclusions:**

Federated learning was used to train the ICD-10 classification model on multicenter clinical text while protecting data privacy. The model’s performance was better than that of models that were trained locally.

## Introduction

### Background

**Figure 1:**
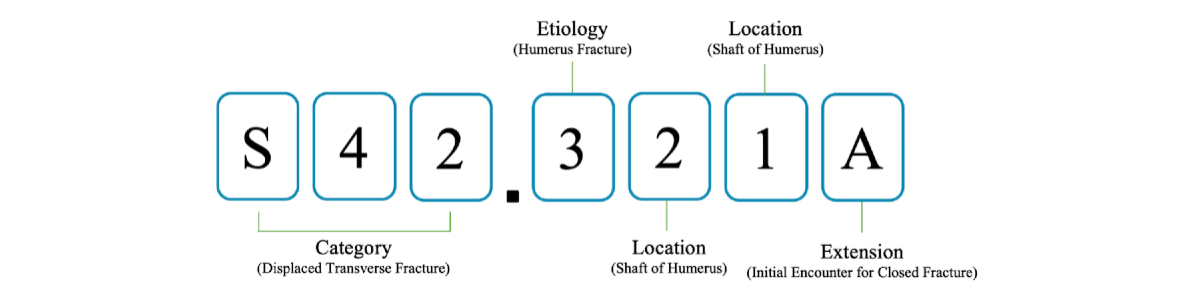
Structure of an *International Classification of Diseases, 10th Revision, Clinical Modification* code.

The World Health Organization published a unified classification system for diagnoses of diseases called the *International Classification of Diseases* (ICD), and the ICD 10th Revision (ICD-10) is widely used [1]. Coders classify diseases according to the rules of the ICD, and the resulting ICD codes are used for surveys, statistics, and reimbursements. The ICD-10 Clinical Modification (ICD-10-CM) is used for coding medical diagnoses and includes approximately 69,000 codes [2,3]. ICD-10-CM codes contain 7 digits; the structure is shown in Figure 1.

In hospitals, diagnoses for each patient are first written as text descriptions in the electronic health record. A coder then reads these records to classify diagnoses into ICD codes. Because diagnoses are initially written as free text, the text's ambiguity makes diagnoses difficult to code. Classifying each diagnosis is very time-consuming. A discharge record may contain 1 to 20 codes. Per the estimation of a trial, coders spent 20 minutes assigning codes to each patient on average [4]. An automatic tool can be used to increase the efficiency of and reduce the labor for ICD classification.

### Related Work

Recently, deep learning and natural language processing (NLP) models have been developed to turn plain text into vectors, making it possible to automatically classify them. Shi et al [5] proposed a hierarchical deep learning model with an attention mechanism. Sammani et al [6] introduced a bidirectional gated recurrent unit model to predict the first 3 or 4 digits of ICD codes based on discharge letters. Wang et al [7] proposed a convolutional neural network model with an attention mechanism and gated residual network to classify Chinese records into ICD codes. Makohon et al [8] showed that deep learning with an attention mechanism effectively enhances ICD-10 predictions. Previous studies also mentioned the necessity of enormous data sets and how privacy-sensitive clinical data limited the development of models for automatic ICD-10 classification [6].

Federated learning has achieved impressive results in the medical field, being used to train models on multicenter data while keeping them private. Federated learning is widely used in medical image and signal analyses, such as brain imaging analysis [9] and the classification of electroencephalography signals [10]. In the clinical NLP field, Liu et al [11] proposed a 2-stage federated method that involved using clinical notes from different hospitals to extract phenotypes for medical tasks.

Previously, we applied a Word2Vec model with a bidirectional gated recurrent unit to classify ICD-10-CM codes from electronic medical records [12]. We analyzed the distribution of ICD-10-CM codes and extracted features from discharge notes. The model had an *F*_1_ score of 0.625 for ICD-10-CM code classification. To improve the model’s performance, we implemented bidirectional encoder representations from transformers (BERT) and found an improved *F*_1_ score of 0.715 for ICD-10-CM code classification [4]. We also found that the coding time decreased when coders used classification model aids; the median *F*_1_ score significantly improved from 0.832 to 0.922 (*P*<.05) in a trial [4]. Furthermore, we constructed a system to improve ease of use, comprising data processing, feature extraction, model construction, model training, and a web service interface [4]. Lastly, we included a rule-based algorithm in the preprocessing process and improved the *F*_1_ score to 0.853 for ICD-10-CM classification [13].

### Objective

This study aims to further improve the performance of the ICD-10 classification model and enable the model’s use across hospitals. In this study, we investigated the effect of federated learning on the performance of a model that was trained on medical text requiring ICD-10 classification.

## Methods

### Ethics Approval

The study protocol was approved by the institutional review boards of Far Eastern Memorial Hospital (FEMH; approval number: 109086-F), National Taiwan University Hospital (NTUH; approval number: 201709015RINC), and Taipei Veterans General Hospital (VGHTPE; approval number: 2022-11-005AC), and the study adhered to the tenets of the Declaration of Helsinki. Informed consent was not applicable due to the use of deidentified data.

### Data Collection

Our data were acquired from electronic health records at FEMH (data recorded between January 2018 and December 2020), NTUH (data recorded between January 2016 and July 2018), and VGHTPE (data recorded between January 2018 and December 2020). The data contained the text of discharge notes and ICD-10-CM codes. Coders in each hospital annotated the ground truth ICD-10 codes.

### Data Description

**Figure 2:**
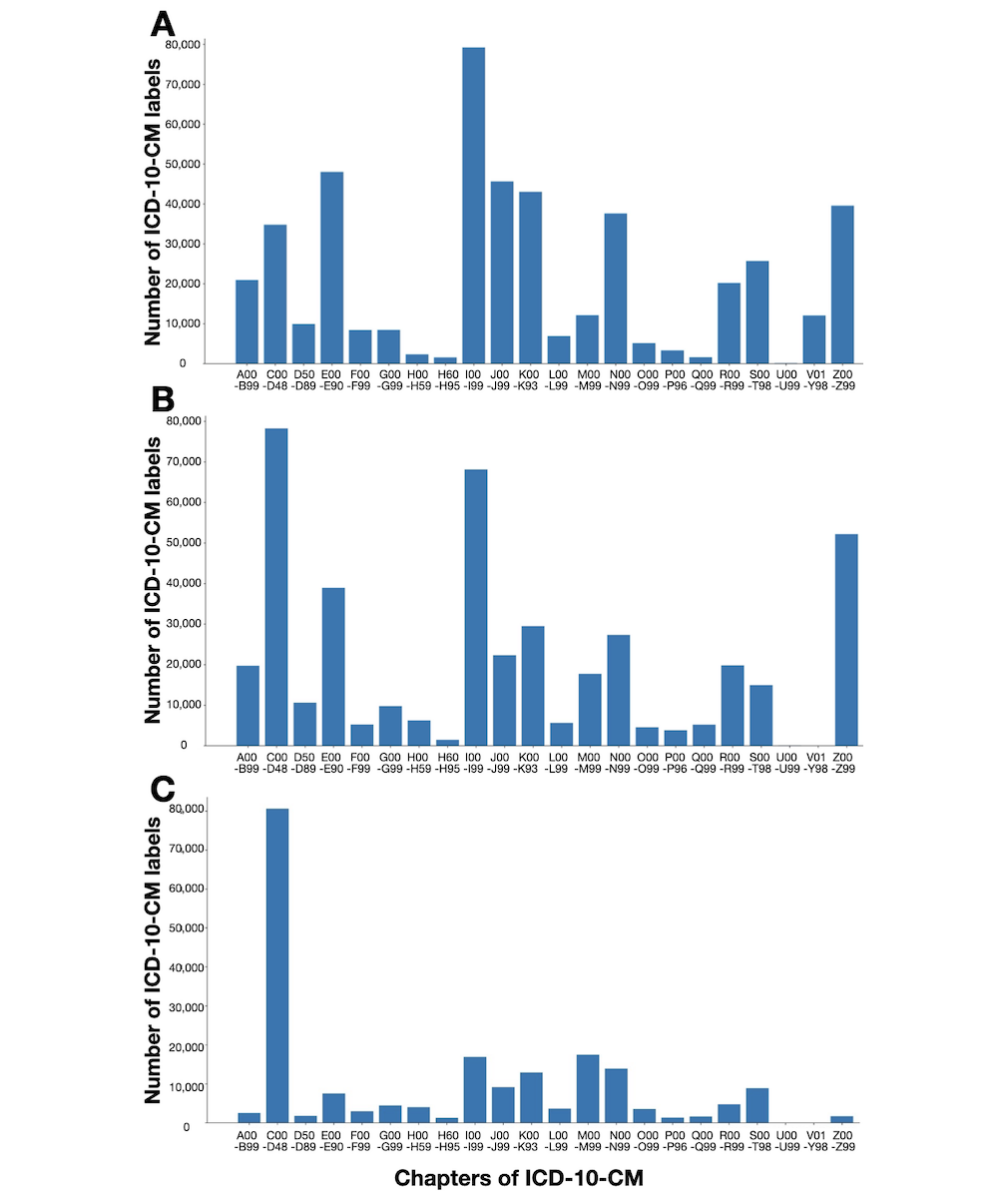
Counts of ICD-10-CM labels for 22 chapters from (A) Far Eastern Memorial Hospital, (B) National Taiwan University Hospital, and (C) Taipei Veterans General Hospital. ICD-10-CM: *International Classification of Diseases, 10th Revision, Clinical Modification*.

After duplicate records were removed, our data set contained 100,334, 239,592, and 283,535 discharge notes from FEMH, NTUH, and VGHTPE, respectively. Each record contained between 1 and 20 ICD-10-CM labels. The distribution of labels for each chapter is shown in Figure 2. These chapters are classified by the first three digits. Codes for chapters V01 to Y98 are not used for insurance reimbursement; hence, they were excluded from our data set. The minimum number of ICD-10-CM labels was found for chapters U00 to U99, and the maximum number was found for chapters J00 to J99. Counts of ICD-10-CM labels from the three hospitals are shown in Multimedia Appendix 1.

The text in the data set contained alphabetic characters, punctuation, and a few Chinese characters. The punctuation count and the top 10 Chinese characters are shown in Multimedia Appendix 2. The most common punctuation mark was the period (“.”), and the least common was the closing brace (“}”).

### Preprocessing

We first removed duplicate medical records from the data set. We then transformed all full-width characters into half-width characters and all alphabetic characters into lowercase letters. Records shorter than 5 characters were removed, as these were usually meaningless words, such as “nil” and “none.” We also removed meaningless characters, such as newlines, carriage returns, horizontal tabs, and formed characters (“\n,” “\r,” “\t,” and “\f,” respectively). Finally, all text fields were concatenated.

To choose a better method for managing punctuation and Chinese characters during the preprocessing stage, we determined model performance by using FEMH data, given the inclusion of these characters in the data. Each experiment used 2 versions of the data. In the first version, we retained these specific characters, and in the second, we removed them. Experiment P investigated the effect of punctuation, experiment C investigated the effect of Chinese characters, and experiment PC investigated the effects of both punctuation and Chinese characters. Another method of retaining Chinese character information is using English translations of Chinese characters. Therefore, we also compared the model’s performance when Chinese characters were retained to its performance when Google Translate was used to obtain English translations.

One-hot encoding was used for the labels. Of the 69,823 available ICD-10-CM codes, 17,745 appeared in our combined data set, resulting in a one-hot encoding vector length of 17,745. The final cohort comprised 100,334, 239,592, and 283,535 records from FEMH, NTUH, and VGHTPE, respectively; 20% (FEMH: 20,067/100,334; NTUH: 47,918/239,592; VGHTPE: 56,707/283,535) of the records were randomly selected for the testing set, and the remaining records were used as the training set.

### Classification Model

We compared the performance of different variants of BERT, including PubMedBERT [14], RoBERTa (Robustly Optimized BERT Pretraining Approach) [15], ClinicalBERT [16], and BioBERT (BERT for Biomedical Text Mining) [17]. BioBERT was pretrained with text from PubMed—the most popular bibliographic database in the health and medical science fields. ClinicalBERT was pretrained with the MIMIC-III (Medical Information Mart for Intensive Care III) data set, and its vocabulary was from English Wikipedia and the BookCorpus data set. PubMedBERT is another variant of BERT that uses training data from PubMed. The main difference between PubMedBERT and BioBERT is their vocabularies. The vocabulary of BioBERT was from English Wikipedia and the BookCorpus data set—as was the vocabulary of BERT—whereas that of PubMedBERT was from PubMed. This difference in vocabularies affects the ability to recognize words in clinical text. RoBERTa used the original BERT model, but it also used a longer training time, a larger batch size, and more training data. The training data were from the BookCorpus, CC-News (CommonCrawl News), and OpenWebText data sets. RoBERTa also applied dynamic masking, which meant that the masked tokens would be changed multiple times instead of being fixed in the original BERT. The vocabularies and corpora of these BERT variants are summarized in Table 1.

**Figure 3:**
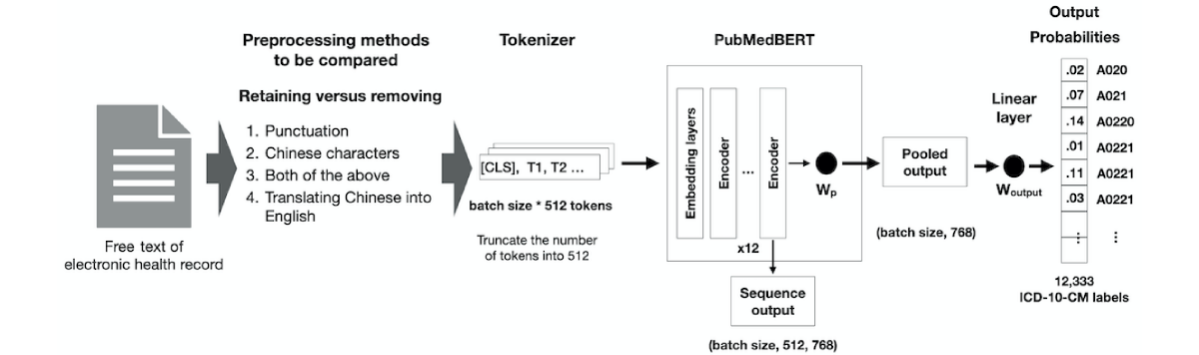
Model architecture and processing flowchart. CLS: class token; ICD-10-CM: *International Classification of Diseases, 10th Revision, Clinical Modification*.

For our comparison, the text was first fed into the BERT tokenizer, which transformed strings into tokens. The number of tokens was then truncated to 512 for every text datum that met the input length limit of 512. A linear layer connected the word embeddings produced from the models to the output layers of the one-hot–encoded multilabels. The output size of the linear layer was 17,745, which matched the one-hot encoding vector size of the labels. Binary cross-entropy was used to calculate the model loss. We trained our model for 100 epochs, with a learning rate of 0.00005. These models were fine-tuned for our ICD-10-CM multilabel classification task to compare their performance. Figure 3 summarizes the model architecture and preprocessing flowchart. The best-performing model and preprocessing method were chosen for subsequent federated learning.

**Table 1:** Summary of the vocabulary and corpus sources for the various bidirectional encoder representations from transformers (BERT) models.

Models	Vocabulary sources	Corpus sources (training data)
PubMedBERT	PubMed	PubMed
RoBERTa^a^	The BookCorpus, CC-News^b^, and OpenWebText data sets	The BookCorpus, CC-News, and OpenWebText data sets
ClinicalBERT	English Wikipedia and the BookCorpus data set	The MIMIC-III^c^ data set
BioBERT^d^	English Wikipedia and the BookCorpus data set	PubMed

^a^RoBERTa: Robustly Optimized BERT Pretraining Approach.

^b^CC-News: CommonCrawl News.

^c^MIMIC-III: Medical Information Mart for Intensive Care III.

^d^BioBERT: BERT for Biomedical Text Mining.

### Federated Learning

With federated learning, a model can be trained without sharing data [18]. Clients (ie, local machines) keep their training data on the same model architecture while exchanging the weights of model parameters. A server receives the weights from each client and averages their weights. After updating the model, the server sends new weights back to the clients. The clients can then start a new training round. We updated the weights of our model parameters with the *FederatedAveraging* algorithm [18] and used Flower for federated learning [19].

Flower is an open-source federated learning framework for researchers [19]. Flower has a server-client structure. The server and clients need to be started individually, and a server needs to be assigned to each client. They communicate via the open-source Google Remote Procedure Call (gRPC; Google LLC) [20]. With the gRPC, a client application can directly call a method on a server application, and this can be done on different machines. There is a registration center on the server for managing communication with all clients. There are 3 main modules in the server. The first—a connection management module—maintains all current gRPC connections. On the server, each gRPC corresponds to each client. When a gRPC is established, the register function is triggered to store the clients’ information in an array. If a client initiates a disconnection or the connection times out, the register function will be called to clear the client. The second module—a bridge module—caches the information, regardless of whether the gRPC information from the clients or the server will be stored in the module. However, since the buffer is shared in both directions, it is necessary to use the state transition method to ensure that all of the information in the buffer is the same. There are five states—the *close*, *waiting for client write*, *waiting for client read*, *waiting for server write*, and *waiting for server read* states. The third module—a server handler—manages the traffic between the server and the clients.

**Figure 4:**
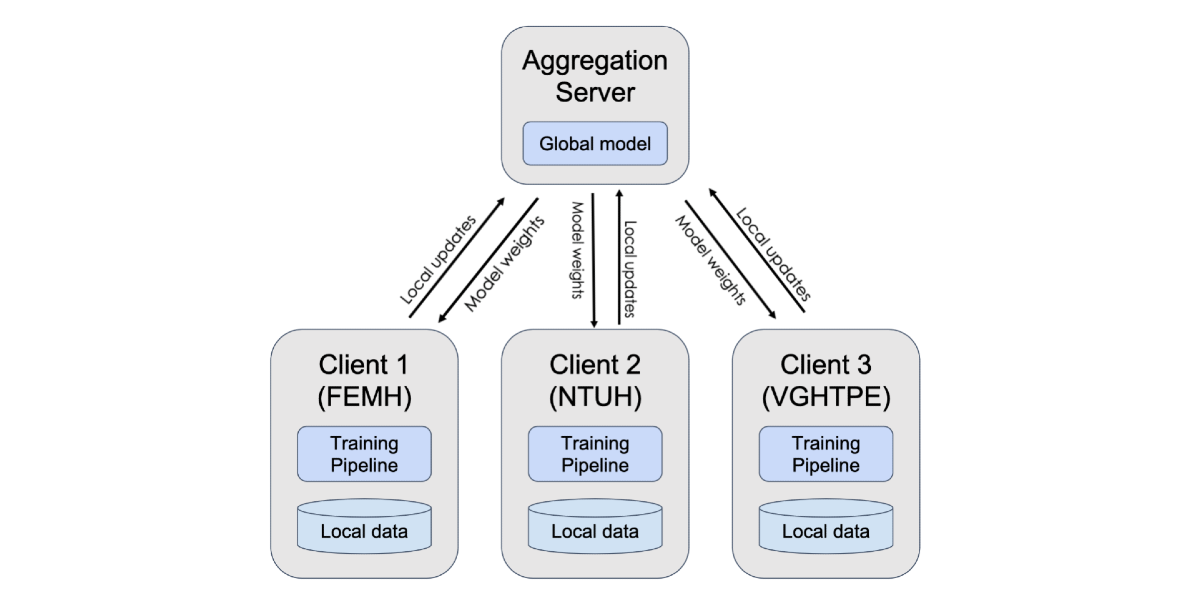
Federated learning architecture. FEMH: Far Eastern Memorial Hospital; NTUH: National Taiwan University Hospital; VGHTPE: Taipei Veterans General Hospital.

Clients were set in the three hospitals, where the model was trained on local data. The weights from each client were transferred to the server, where the weights were averaged, and global models were made (Figure 4). We set 5 epochs for each training round on clients and 20 rounds for the server aggregation. Our study was conducted on 2 nodes. Each node had a NVIDIA RTX 2080 Ti graphics processing unit (NVIDIA Corporation) with 64 GB of RAM, and one node had 2 NVIDIA TITAN RTX graphics processing units with 64 GB of RAM (NVIDIA Corporation).

### Label Attention

**Figure 5:**
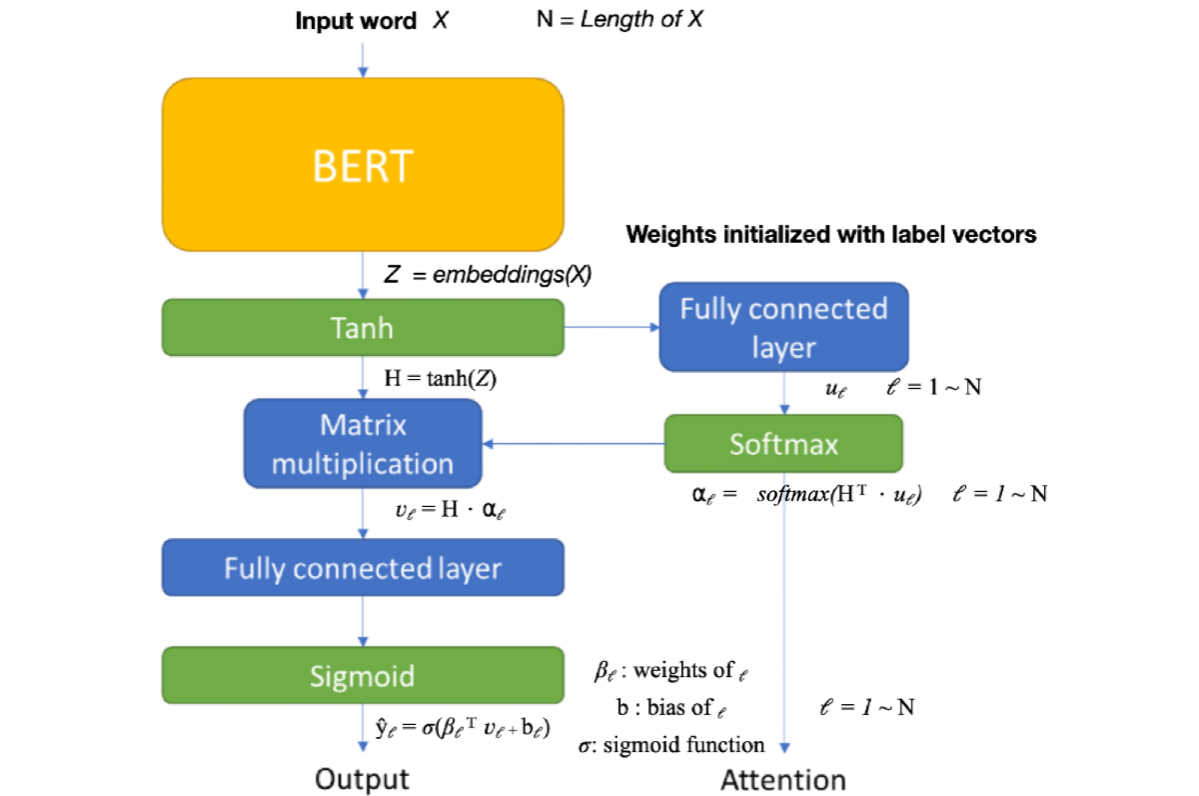
Our model architecture with label attention. BERT: bidirectional encoder representations from transformers.

To explain the outputs of our model, we added a label attention architecture [21]. It calculated the attention based on the inner products of word vectors and each label vector separately. Figure 5 shows how we added the label attention architecture to our model. First, we fine-tuned the BERT model by using the definitions of ICD-10-CM codes to generate the label vectors. Second, we constructed a fully connected layer, of which the weights were initialized with the label vectors. Third, the output produced by BERT was passed through the hyperbolic tangent function, thereby producing word vectors. We inputted the word vectors (Ζ) into the fully connected layer and softmax layer. The output (⍺) of the softmax layer was the attention. Fourth, we inputted the hyperbolic tangent function of word vectors (H), which were multiplied by attention (⍺), into another fully connected layer and sigmoid layer. This was similar to our original architecture. The output (y) could be subtracted from the one-hot–encoded labels for the loss calculation. Finally, attention was used to explain how the model predicted the labels. Attention was given to the input text for corresponding ICD-10-CM codes. The performance of the model after adding the label attention architecture was compared to its performance without this architecture.

### Metrics

We used the micro *F*_1_ score to evaluate performance because it is the harmonic mean of precision and recall and therefore yields more balanced results than those yielded when using precision or recall only. The micro *F*_1_ score was calculated as follows:



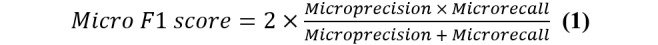



where



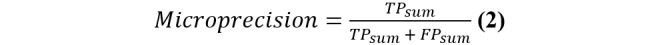



and



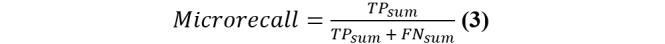



*TP_sum_* indicates the sum of true positives, *FP_sum_* indicates the sum of false positives, and *FN_sum_* indicates the sum of false negatives.

## Results

### Comparing the Performance of Different BERT Models

The *F*_1_ scores of PubMedBERT, RoBERTa, ClinicalBERT, and BioBERT were 0.735, 0.692, 0.711, and 0.721, respectively. The *F*_1_ score of PubMedBERT was the highest, and that of RoBERTa was the lowest among all models (Table 2). Due to these results, we used PubMedBERT in the subsequent experiments.

**Table 2:** Performance of different bidirectional encoder representations from transformers (BERT) models.

Models	*F*_1_ score	Precision	Recall
PubMedBERT	0.735	0.756	0.715
RoBERTa^a^	0.692	0.719	0.666
ClinicalBERT	0.711	0.735	0.689
BioBERT^b^	0.721	0.754	0.691

^a^RoBERTa: Robustly Optimized BERT Pretraining Approach.

^b^BioBERT: BERT for Biomedical Text Mining.

### The Model’s Performance When Retaining or Removing Punctuation or Chinese Characters

Table 3 shows the mean number of tokens for each data set preprocessing case. The mean number of tokens when removing punctuation and Chinese characters was 52.9. The mean number of tokens when the characters were retained in experiment P (punctuation), experiment C (Chinese characters), and experiment PC (punctuation and Chinese characters) was 65.0, 53.1, and 65.1, respectively. Punctuation and Chinese characters comprised 18.3% (1,301,988/7,096,460) and 0.1% (7948/7,096,460) of the tokens in our data, respectively.

**Table 3:** Mean number of data tokens for retaining or removing punctuation or Chinese characters.

Experiment	Mean number of tokens
Removed punctuation and Chinese characters (baseline)	52.9
Retained punctuation	65.0
Retained Chinese characters	53.1
Retained punctuation and Chinese characters	65.1

Table 4 shows the *F*_1_ scores for each data set preprocessing case. The baseline performance of the model after removing punctuation and Chinese characters was 0.7875. In experiment P, the *F*_1_ score for retaining punctuation was 0.8049—an increase of 0.0174 (2.21%). In experiment C, the *F*_1_ score for retaining Chinese characters was 0.7984—an increase of 0.0109 (1.38%). In experiment PC, the *F*_1_ score for retaining punctuation and Chinese characters was 0.8120—an increase of 0.0245 (3.11%). In all experiments, retaining these characters was better than removing them, with experiment PC showing the largest improvement in performance.

**Table 4:** *F*_1_ scores for retaining or removing punctuation or Chinese characters.

Experiment	*F*_1_ score	Absolute increases (percentage)
Removed punctuation and Chinese characters (baseline)	0.7875	N/A^a^
Retained punctuation	0.8049	0.0174 (2.21%)
Retained Chinese characters	0.7984	0.0109 (1.38%)
Retained punctuation and Chinese characters	0.8120	0.0245 (3.11%)

^a^N/A: not applicable.

### The Model’s Performance Before and After Translation

In the experiment where we translated Chinese into English, the *F*_1_ score for retaining the Chinese characters was 0.7984, and that for translating them into English was 0.7983.

### Federated Learning

Table 5 shows the performance of the models that were trained in the three hospitals. The models trained in FEMH, NTUH, and VGHTPE had validation *F*_1_ scores of 0.7802, 0.7718, and 0.6151, respectively. The FEMH model had testing *F*_1_ scores of 0.7412, 0.5116, and 0.1596 on the FEMH, NTUH, and VGHTPE data sets, respectively. The NTUH model had testing *F*_1_ scores of 0.5583, 0.7710, and 0.1592 on the FEMH, NTUH, and VGHTPE data sets, respectively. The VGHTPE model had testing *F*_1_ scores of 0.1081, 0.1058, and 0.5692 on the FEMH, NTUH, and VGHTPE data sets, respectively. The weighted average testing *F*_1_ scores were 0.4472, 0.5353, and 0.2522 for the FEMH, NTUH, and VGHTPE models, respectively.

Table 6 shows the federated learning model’s performance in the three hospitals. The federated learning model had validation *F*_1_ scores of 0.7464, 0.6511, and 0.5979 on the FEMH, NTUH, and VGHTPE data sets, respectively. The federated learning model had testing *F*_1_ scores of 0.7103, 0.6135, and 0.5536 on the FEMH, NTUH, and VGHTPE data sets, respectively. The weighted average testing *F*_1_ score was 0.6142 for the federated learning model.

**Table 5:** Models that were trained in the three hospitals for *International Classification of Diseases, 10th Revision* classification.

Hospitals	Validation *F*_1_ score	Testing *F*_1_ scores	Weighted average testing *F*_1_ scores
FEMH^a^	0.7802	0.7412 (FEMH) 0.5116 (NTUH^b^) 0.1596 (VGHTPE^c^)	0.4472
NTUH	0.7718	0.5583 (FEMH) 0.7710 (NTUH) 0.1592 (VGHTPE)	0.5353
VGHTPE	0.6151	0.1081 (FEMH) 0.1058 (NTUH) 0.5692 (VGHTPE)	0.2522

^a^FEMH: Far Eastern Memorial Hospital.

^b^NTUH: National Taiwan University Hospital.

^c^VGHTPE: Taipei Veterans General Hospital.

**Table 6:** The federated learning model’s performance in the three hospitals.

Data	Validation *F*_1_ score	Testing *F*_1_ score^a^
FEMH^b^ data	0.7464	0.7103
NTUH^c^ data	0.6511	0.6135
VGHTPE^d^ data	0.5979	0.5536

^a^The weighted average testing *F*_1_ score was 0.6142.

^b^FEMH: Far Eastern Memorial Hospital.

^c^NTUH: National Taiwan University Hospital.

^d^VGHTPE: Taipei Veterans General Hospital.

### Label Attention

The *F*_1_ scores of the model with and without the label attention mechanism were 0.804 (precision=0.849; recall=0.763) and 0.813 (precision=0.852; recall=0.777), respectively.

**Figure 6:**
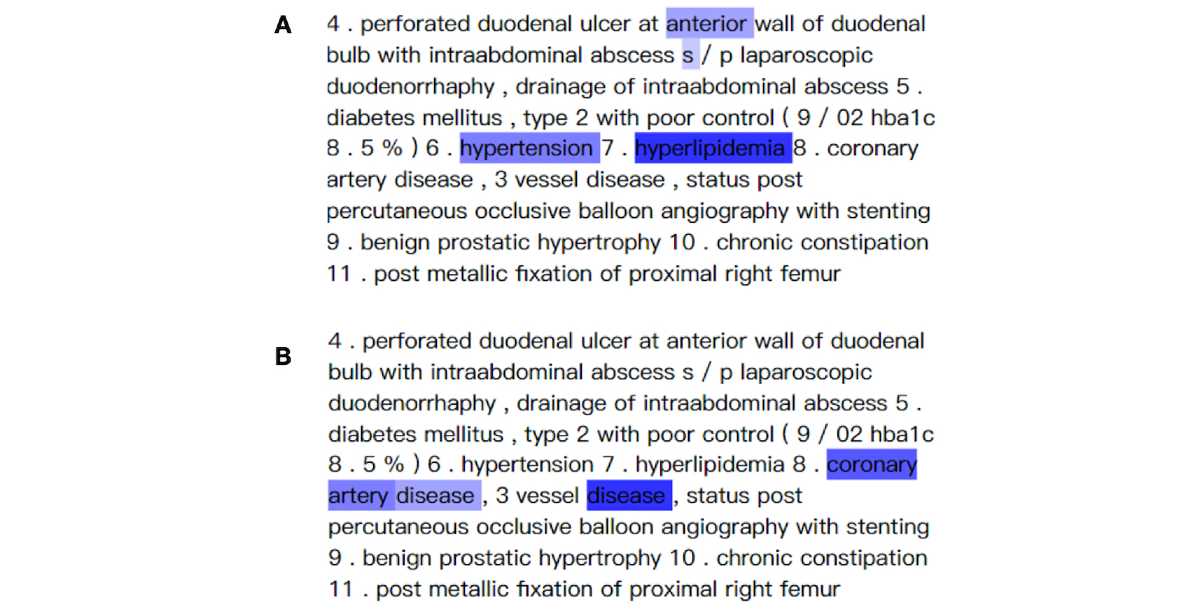
Attention for *International Classification of Diseases, 10th Revision, Clinical Modification* codes (A) E78.5 (“Hyperlipidemia, unspecified”) and (B) I25.10 (“Atherosclerotic heart disease of native coronary artery without angina pectoris”). The intensity of the blue color represents the magnitude of the attention score.

Figure 6 shows a visualization of the attention for ICD-10-CM codes and their related input text. The words were colored blue based on the attention scores for different labels. The intensity of the blue color represented the magnitude of the attention score. We used ICD-10-CM codes E78.5 (“Hyperlipidemia, unspecified”) and I25.10 (“Atherosclerotic heart disease of native coronary artery without angina pectoris”) as examples.

## Discussion

### Principal Findings

The federated learning model outperformed each local model when tested on external data. The weighted average *F*_1_ scores on the testing set were 0.6142, 0.4472, 0.5353, and 0.2522 for the federated learning, FEMH, NTUH, and VGHTPE models, respectively (Table 5 and Table 6). The model’s performance decreased when tested on external data. Because different doctors, coders, and diseases are found in different hospitals, the style of clinical notes may be distinct across hospitals. Overcoming such gaps among hospitals is challenging. Although the performance of the federated learning model was inferior to that of the models trained on local data when tested on local data, its performance was higher than that of the models trained on local data when tested on external data. Moreover, in the VGHTPE data set, the label distribution was very different from the label distributions in the other two hospitals’ data sets (Figure 2). Therefore, the VGHTPE model only achieved *F*_1_ scores of 0.1058 and 0.1081 on the NTUH and FEMH testing sets, respectively. The FEMH and NTUH models had *F*_1_ scores of 0.1596 and 0.1592, respectively, on the VGHTPE testing set (Table 5).

Federated learning improves model performance on external data. Federated learning can be used to build an ICD coding system for use across hospitals. However, the training time required for federated learning is longer than the training time required for local deep learning. Federated learning takes approximately 1 week, and local training takes approximately 2 days. There are 2 reasons for this. First, the communication between the server and the clients takes longer if the model is large. The size of our model is approximately 859 MB. Second, different clients may have different computing powers, and the slower client becomes a bottleneck [22,23]. Other clients may wait for the slower client until it completes its work.

The performance of PubMedBERT was better than that of BioBERT, ClinicalBERT, and RoBERTa. Table 2 shows that the vocabulary of BERT models is an important factor of model performance. The vocabulary of PubMedBERT contains predominantly medical terms, whereas the vocabularies of the other three models contain common words. This difference affects the ability to recognize words in clinical text. Most published BERT models use a vocabulary of 30,522 WordPieces [24]. However, these vocabulary data do not contain some words from special fields. For example, the medical term “lymphoma” is in the vocabulary of PubMedBERT but not in the vocabularies of BioBERT, ClinicalBERT, and RoBERTa. The term “lymphoma” can be transformed into the token “lymphoma” by the PubMedBERT tokenizer, but the term would be split into 3 tokens—“l”, “##ymph”, and “##oma”—by BioBERT, ClinicalBERT, and RoBERTa.

In most scenarios, nonalphanumeric characters are removed because they are considered useless to the models [25]. In contrast to models with attention mechanisms, early NLP models could not pay attention to punctuation. Additional characters would make the models unable to focus well on keywords. The removal of punctuation in English text and text in other languages, such as Arabic, has been performed for NLP [26]. Ek et al [27] compared 2 data sets of daily conversation text—one retained punctuation, and the other did not. Their results showed better performance for the data set that retained punctuation.

For experiments P, C, and PC, all models performed better when additional characters were retained (Table 4). Experiment P demonstrated that PubMedBERT could use embedded punctuation. As punctuation marks are used to separate different sentences, removing them connects all sentences and thus makes it harder for a model to understand the text content. The improvement in our *F*_1_ score for retaining punctuation is similar to the results of previous work by Ek et al [27]. Our results demonstrate that retaining punctuation can improve the performance of text classification models for text from the clinical field. Experiment C demonstrated that PubMedBERT could use embedded Chinese characters. Although PubMedBERT was pretrained with mostly English text, its vocabulary contains many Chinese characters. The tokens from Chinese characters may contribute to the ICD-10 classification task for clinical text because they provide information such as place names, trauma mechanisms, and local customs. The results of experiment PC indicate that the benefits of retaining punctuation and retaining Chinese characters are additive. In the translation experiment, the *F*_1_ scores did not considerably differ. This result indicates that the model can extract information from clinical text in either English or Chinese. The use of the attention mechanisms of BERT increased our model’s ability to pay attention to keywords. Punctuation and Chinese characters contribute helpful information to these models. Therefore, this preprocessing strategy—retaining more meaningful tokens—provides more information for ICD-10 classification task models.

In our previous study, we introduced an attention mechanism to visualize the attention given to the input text for ICD-10 definitions [4]. Through this approach, we trained a model to predict ICD-10 codes and trained another model to extract attention data. This approach might result in inconsistencies between the predictions and attention. In this study, we introduced the label attention architecture to visualize the attention given to the input text for ICD-10 codes [21]. This method better illustrated the attention given to the input words that were used to predict ICD codes, as it is consistent with the methods used by prediction models.

The *F*_1_ score of the model, after the label attention mechanism was added, decreased by 0.009. Although the *F*_1_ score decreased, we obtained explainable predictions. For ICD-10-CM codes E78.5 (“Hyperlipidemia, unspecified”) and I25.10 (“Atherosclerotic heart disease of native coronary artery without angina pectoris”), our model successfully paid great attention to the related words “hyperlipidemia” and “coronary artery” (Figure 6). Our visualization method (ie, highlighting input words) allows users to understand how our model identified ICD-10-CM codes from text.

### Limitations

Our study has several limitations. First, our data were acquired from 3 tertiary hospitals in Taiwan. The extrapolation of our results to hospitals in other areas should be studied in the future. Second, although our results suggest that model performance is better when punctuation and Chinese characters are retained, this effect may be restricted to specific note types. This finding should be further examined in the context of classifying other types of clinical text. Third, the translated text in our last experiment may not be as accurate as translations by a native speaker. However, it is difficult to manually translate large amounts of data. As such, we could only automatically translate the text by using Google Translate.

It should be noted that there is a primary and secondary diagnosis code for each discharge note. Although choosing the primary code makes reimbursements different, the model proposed in this study did not identify primary codes. To make our model capable of identifying a primary code, we proposed a sequence-to-sequence model in our previous work [4]. It transforms the original predicted labels that were concatenated alphabetically, so that they are ordered by diagnosis code. This structure can be added to the model proposed in this study. Predictions based on primary and secondary diagnosis codes can further improve the usability of this system.

### Conclusions

Federated learning was used to train the ICD-10 classification model on multicenter clinical text while protecting data privacy. The model’s performance was better than that of models that were trained locally. We showed the explainable predictions by highlighting input words via a label attention architecture. We also found that the PubMedBERT model can use the meanings of punctuation and non-English characters. This finding demonstrates that changing the preprocessing method for ICD-10 multilabel classification can improve model performance.

## Appendix

Multimedia Appendix 1 Counts of ICD-10-CM labels from the three hospitals. (A) Ranking of counts of labels in a medical record. (B) Ranking of counts of ICD-10-CM codes. ICD-10-CM: *International Classification of Diseases, 10th Revision, Clinical Modification*.

Multimedia Appendix 2 The punctuation count and the top 10 Chinese characters.

## Abbreviations

BERT: bidirectional encoder representations from transformers
BioBERT: Bidirectional Encoder Representations From Transformers for Biomedical Text Mining
CC-News: CommonCrawl News
FEMH: Far Eastern Memorial Hospital
gRPC: Google Remote Procedure Call
ICD-10: *International Classification of Diseases, 10th Revision*
ICD-10-CM: *International Classification of Diseases, 10th Revision, Clinical Modification*
ICD: *International Classification of Diseases*
MIMIC-III: Medical Information Mart for Intensive Care III
NLP: natural language processing
NTUH: National Taiwan University Hospital
RoBERTa: Robustly Optimized Bidirectional Encoder Representations From Transformers Pretraining Approach
VGHTPE: Taipei Veterans General Hospital
