# Supplementary material for: Training a Deep Contextualized Language Model for International Classification of Diseases, 10th Revision Classification via Federated Learning: Model Development and Validation Study
Source: JMIR Med Inform. 2022 Nov 10;10(11):e41342. doi: 10.2196/41342 (PMC9693720; doi:10.2196/41342)

Figure S1. Counts of ICD-10-CM labels in Far Eastern Memorial Hospital. (A) Ranking of counts of labels in a medical record; (B) Ranking of counts of ICD-10-CM codes.

(A)


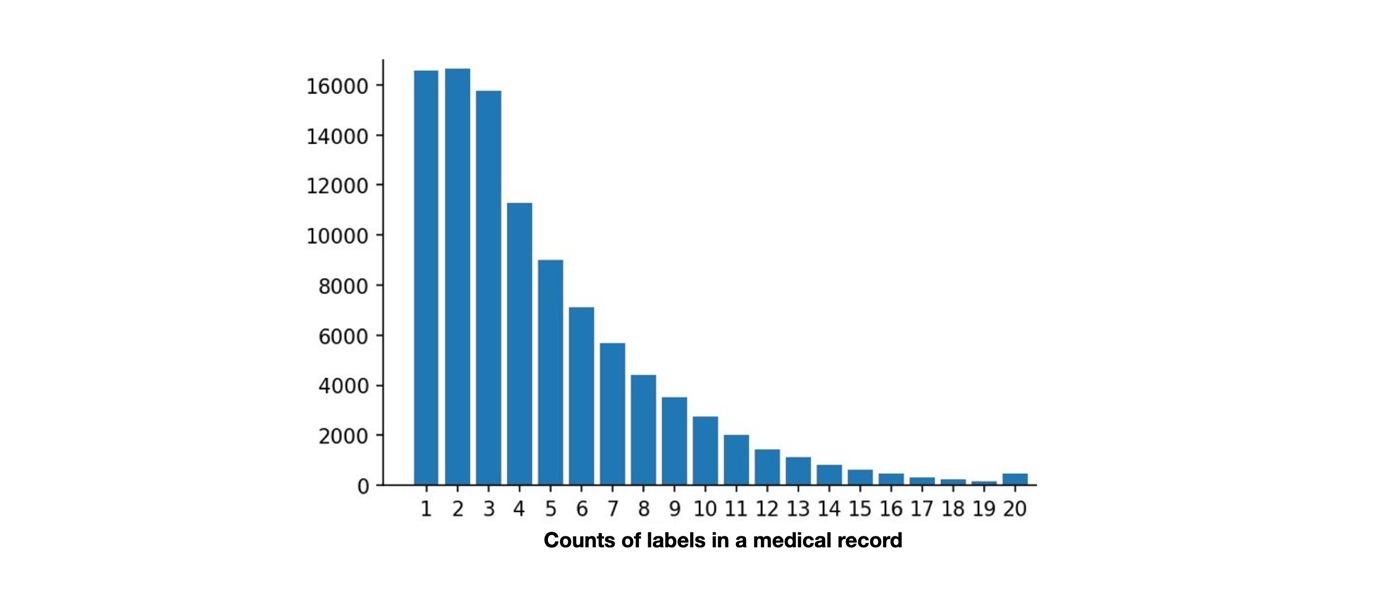


(B)


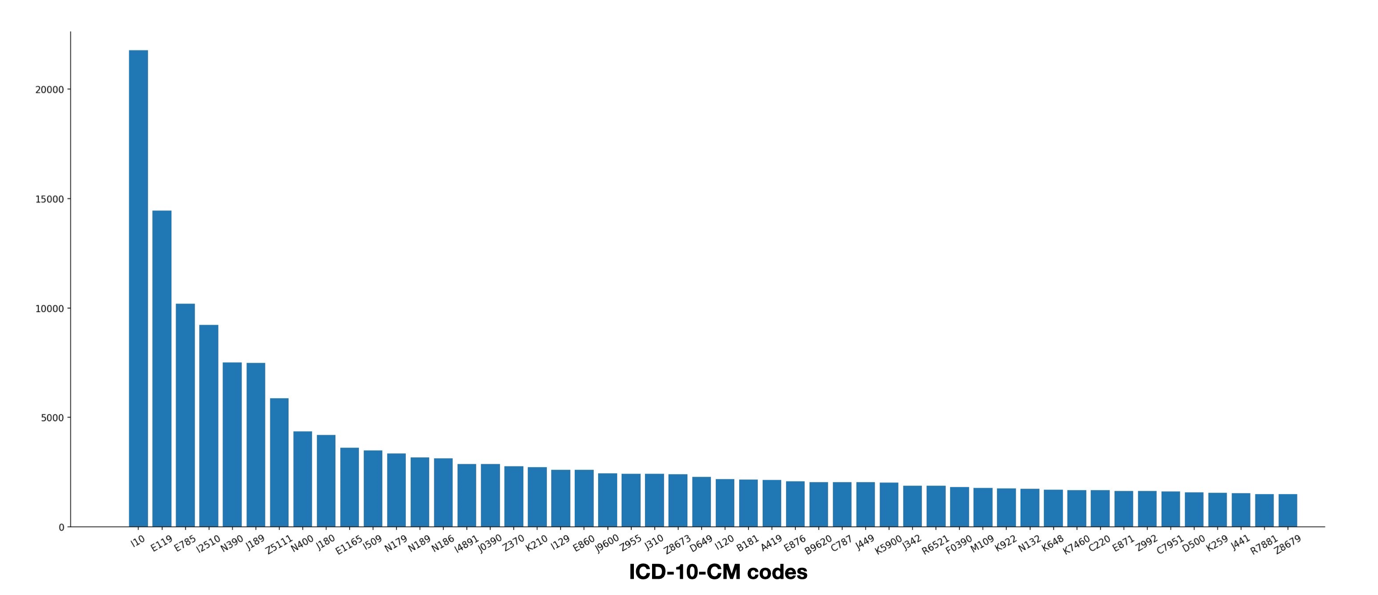


Figure S2. Counts of ICD-10-CM labels in National Taiwan University Hospital. (A) Ranking of counts of labels in a medical record; (B) Ranking of counts of ICD-10-CM codes.

(A)


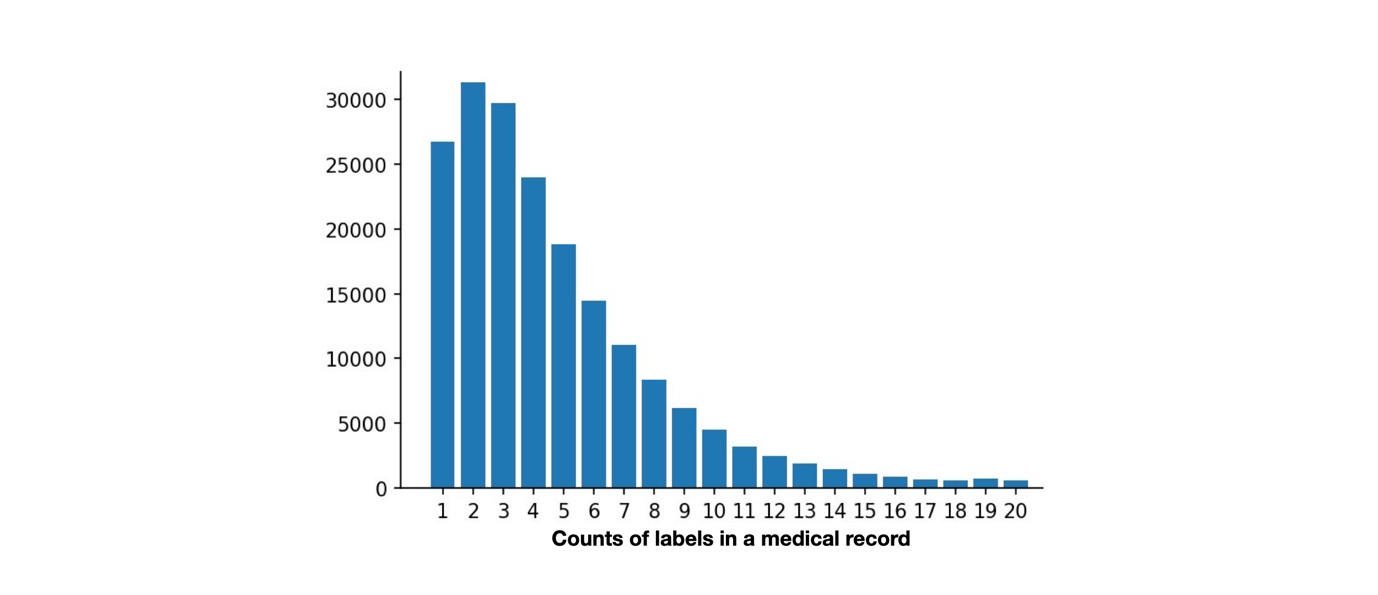


(B)


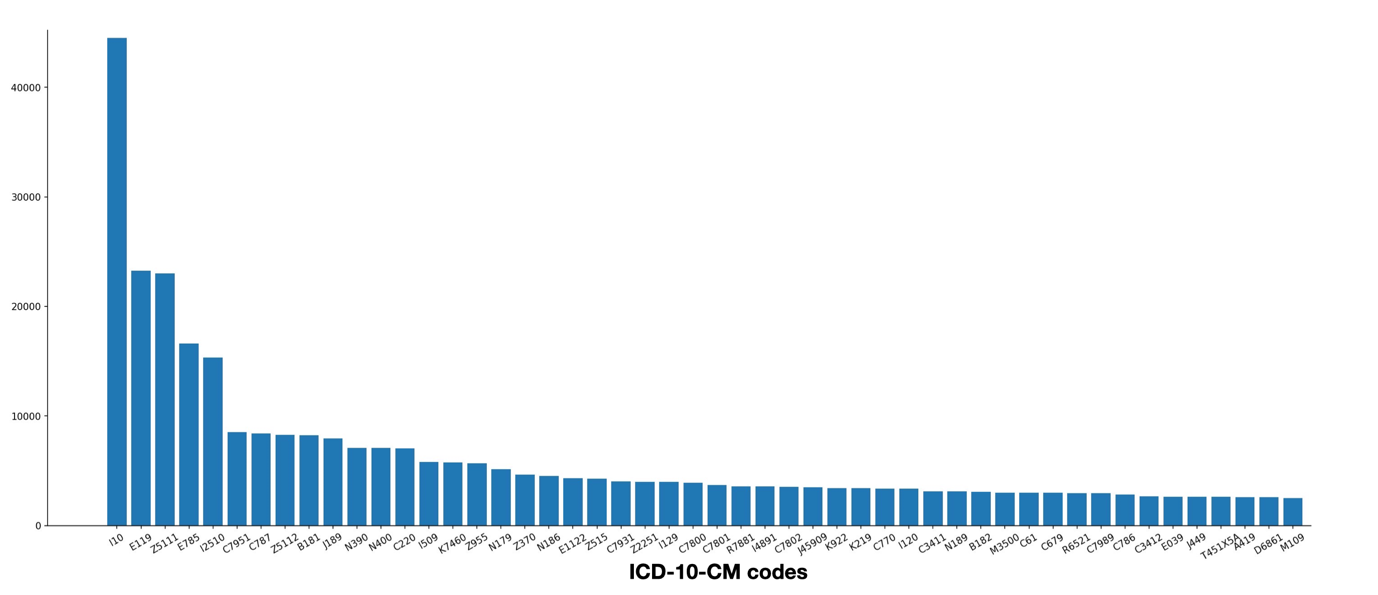


Figure S3. Counts of ICD-10-CM labels in Taipei Veterans General Hospital. (A) Ranking of counts of labels in a medical record; (B) Ranking of counts of ICD-10-CM codes.

(A)


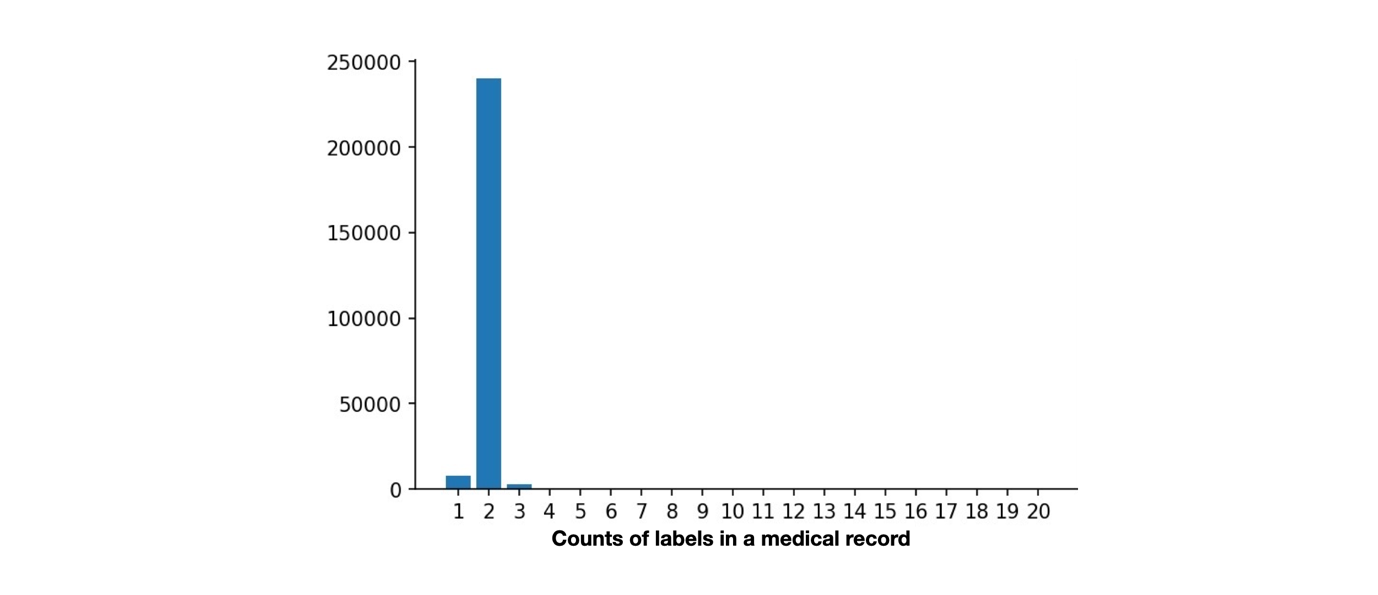


(B)


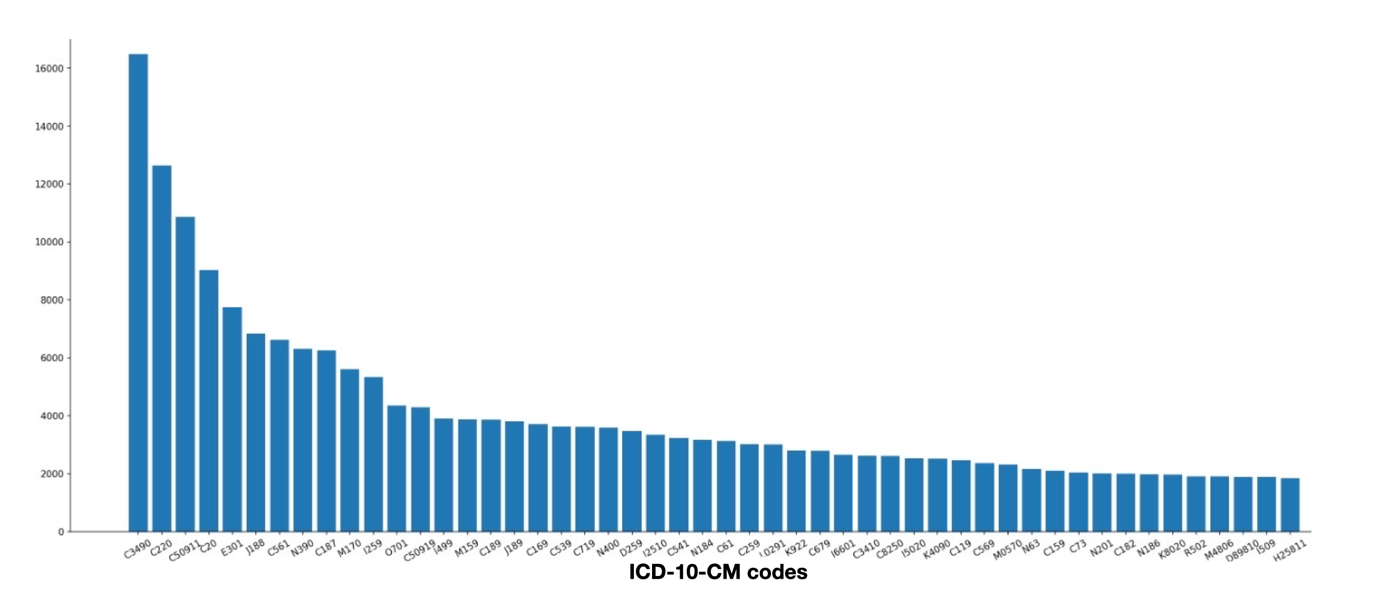

Supplement: Multimedia Appendix 1 [file medinform_v10i11e41342_app1.docx]
