# Supplementary material for: Training a Deep Contextualized Language Model for International Classification of Diseases, 10th Revision Classification via Federated Learning: Model Development and Validation Study
Source: JMIR Med Inform. 2022 Nov 10;10(11):e41342. doi: 10.2196/41342 (PMC9693720; doi:10.2196/41342)
Supplement: Multimedia Appendix 2 [file medinform_v10i11e41342_app2.docx]

**Table S1.** Punctuation count in our data

| **Punctuation mark** | **Count** |
| --- | --- |
| . | 444,848 |
| , | 378,231 |
| / | 227,027 |
| - | 207,169 |
| : | 78,064 |
| ) | 76,001 |
| ( | 71,788 |
| + | 19,423 |
| ; | 14,046 |
| % | 9,052 |
| * | 8,182 |
| ~ | 5,636 |
| ` | 5,602 |
| > | 5,000 |
| = | 3,672 |
| & | 2,931 |
| ' | 2,457 |
| # | 2,350 |
| < | 1,811 |
| [ | 1,698 |
| ] | 1,689 |
| ? | 1,043 |
| _ | 399 |
| " | 104 |
| $ | 41 |
| ^ | 28 |
| ! | 14 |
| @ | 14 |
| \ | 10 |
| { | 3 |
| \| | 1 |

**Table S2.** Top 10 Chinese characters in our data

| **Chinese character** | **Count** |
| --- | --- |
| 醫 | 473 |
| 院 | 460 |
| 以 | 415 |
| 可 | 341 |
| 否 | 328 |
| 抹 | 279 |
| 片 | 279 |
| 難 | 246 |
| 判 | 245 |
| 讀 | 245 |
| The others | 12,749 |
